# Tumorigenic potential of circulating prostate tumor cells

**DOI:** 10.18632/oncotarget.895

**Published:** 2013-03-05

**Authors:** Filipe LF. Carvalho, Brian W. Simons, Emmanuel S. Antonarakis, Zeshaan Rasheed, Nora Douglas, Daniela Villegas, William Matsui, David M. Berman

**Affiliations:** ^1^ Department of Pathology, Johns Hopkins University School of Medicine, Baltimore, MD, USA; ^2^ Department of Molecular and Comparative Pathobiology, Johns Hopkins University School of Medicine, Baltimore, MD, USA; ^3^ Department of Oncology, Johns Hopkins University School of Medicine, Baltimore, MD, USA; ^4^ Departments of Pathology and Molecular Medicine and Cancer Biology and Genetics, Cancer Research Institute, Queen's University, Kingston, Ontario, Canada

**Keywords:** Circulating tumor cells, EpCAM, prostate cancer, prostate-specific antigen, TRAMP mouse

## Abstract

Circulating tumor cells (CTCs) have received intense scientific scrutiny because they travel in the bloodstream and are therefore well situated to mediate hematogenous metastasis. However, the potential of CTCs to actually form new tumors has not been tested. Popular methods of isolating CTCs are biased towards larger, more differentiated, non-viable cells, creating a barrier to testing their tumor forming potential. Without relying on cell size or the expression of differentiation markers, our objective was to isolate viable prostate CTCs from mice and humans and assay their ability to initiate new tumors. Therefore, blood was collected from transgenic adenocarcinoma of the mouse prostate (TRAMP) mice and from human patients with metastatic castration-resistant prostate cancer (PCa). Gradient density centrifugation or red cell lysis was used to remove erythrocytes, and then leukocytes were depleted by magnetic separation using CD45 immunoaffinity beads. CTCs fractions from TRAMP mice and PCa patients were verified by immunocytochemical staining for cytokeratin 8 and EpCAM, and inoculated into immunodeficient mice. TRAMP tumor growth was monitored by palpation. Human tumor growth formation was monitored up to 8 months by ultrasensitive PSA assays performed on mouse serum. We found viable tumor cells present in the bloodstream that were successfully isolated from mice without relying on cell surface markers. Two out of nine immunodeficient mice inoculated with TRAMP CTCs developed massive liver metastases. CTCs were identified in blood from PCa patients but did not form tumors. In conclusion, viable CTCs can be isolated without relying on epithelial surface markers or size fractionation. TRAMP CTCs were tumorigenic, so CTCs isolated in this way contain viable tumor-initiating cells. Only two of nine hosts grew TRAMP tumors and none of the human CTCs formed tumors, which suggests that most CTCs have relatively low tumor-forming potential. Future studies should identify and target the highly tumorigenic cells.

## INTRODUCTION

Elevated numbers of circulating tumor cells (CTCs) in several cancers, including prostate cancer (PCa) have been correlated with decrease patient survival [1-4]. Thus, CTCs may be potent mediators of prostate cancer metastasis. However, the biological properties of these cells have not been explored. Current methods to isolate these cells rely on physical properties (large size, density, electric charges, and deformability) and expression of the epithelial markers, mainly the epithelial cell adhesion molecule (EpCAM) [5]. Although these methods enable a molecular profiling of CTCs [6], they have important limitations regarding the selected cells. Cancer cells are variable in size, and larger cancer cells are more differentiated and less adept at forming new tumors [7-9]. Therefore, CTCs isolated on the basis of large size may under-represent the underlying CTC population and bias them towards a less tumorigenic phenotype. Likewise, EpCAM expression is absent in subpopulations of CTCs that may be decisive for metastasis [10]. Gene expression profile of prostate cells varies during disease progression [11, 12]. Indeed, cancer cells that lose epithelial differentiation in favor of more mesenchymal character may contribute to carcinogenesis and be more potent initiators of metastasis [13-15]. Furthermore, upon isolation by current methods, CTCs are dead and therefore not usable in functional experiments. Indeed, at the time this publication was submitted, we could find no other published studies characterizing growth, differentiation potential, clonogenicity, or metastatic capacity of circulating tumor cells from any solid tumor.

An efficient xenografting method can be very useful in characterizing the molecular changes of individual tumors during disease progression and response to therapy [16]. Primary human prostate cancers are notoriously difficult to grow in the laboratory setting. Using optimized methods enhanced by co-engraftment with fetal urogenital mesenchyme, 100,000 or more primary human cancer cells are required to establish a tumor [17], suggesting that most primary human PCa cells lack tumorigenic potential. Some investigators have worked to enhance PCa grafting efficiency by growing primary prostate tumors under the kidney capsule of immunodeficient mice [17-19].

In contrast to primary cancers, advanced metastatic cancers may contain a higher fraction of highly tumorigenic cells. In this study, we utilized blood from men with metastatic castration-resistant PCa and from the transgenic adenocarcinoma of the mouse prostate (TRAMP) mice with metastatic disease. TRAMP is the most widely studied mouse model of prostate cancer, and in contrast to human-derived models, it metastasizes widely. In this model, a prostate-specific probasin promoter drives the expression of simian virus 40 (SV40) T antigen in the prostate. This viral oncogene induces malignant transformation of prostate cells at 12 weeks of age, and by 30 weeks, tumors progress to lung and lymph node metastases [20-22].

Our main goal was to isolate a viable unbiased population of CTCs and generate new tumors to test whether these circulating cancer cells have tumorigenic potential.

## RESULTS

### CTCs from TRAMP mouse produce metastases in immunodeficient host

**Figure 1:**
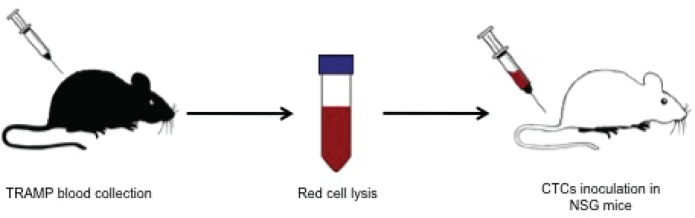
Schematic view of TRAMP CTCs isolation and xenograft TRAMP mice were terminally bleed and the whole blood mixed with red cell lysis buffer. After red cell lysis, the remaining cells were injected in the tail vein of NSG mice.

**Figure 2:**
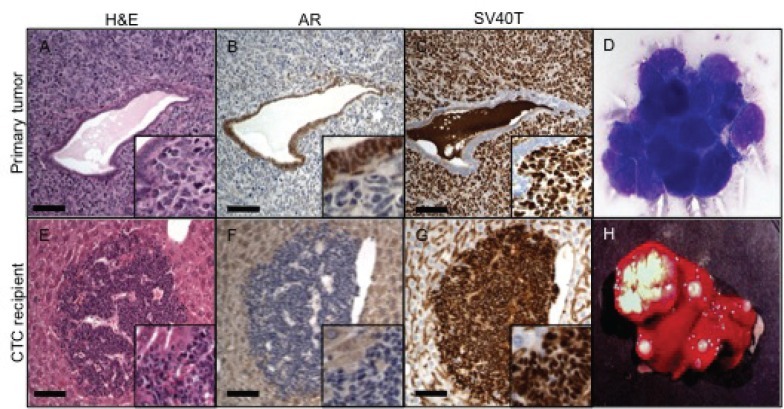
CTCs in the TRAMP mouse *Top row*. H&E stain (A) of primary tumor cells surrounding a benign gland; AR (B) and SV 40 (C) exclusively stain two distinct cell populations: AR the benign cells and SV40 the tumor cells. (D) Large atypical cells found in the bloodstream of TRAMP showing adherent clumps. *Lower row*. Multiple metastasis in the liver of NSG mice. H&E stain of a metastatic lesion near a liver vessel (E). Tumor cells do not express AR (F) and are strongly positive for SV 40 (G). Gross appearance of the liver with massive metastasis (H). Scale bars, 100 μm.

We injected nucleated cell preparations from 700 mL blood (which is approximately 50% of the total blood volume) of TRAMP mice intravenously into nine highly immunodeficient NOD.Cg-*Prkdc*^*scid*^ *Il2rg*^*tm1Wjl*^/SzJ (NSG) mouse recipients (Fig. 1). Three months after injection, seven of the mice showed no evidence of metastasis. The remaining two recipients developed massive tumor deposits in the liver (Fig. 2 E-H). In each mouse, gross inspection of the liver revealed more than 15 liver lesions larger than 1 mm (largest lesion 2cmx1.2cmx1.5cm) and 10 lesions smaller than 1 mm. The lungs, spleen and kidneys did not show evidence of metastatic lesions. Histologic examination of the liver lesions showed tumor cells with neuroendocrine carcinoma morphology typical of TRAMP tumors (Fig. 2 A-D). TRAMP cancers invaded diffusely through the liver parenchyma (Fig. 2 E-H). As seen in previous studies of TRAMP tumors, blood-derived TRAMP tumor cells in the liver lacked immunoreactivity for androgen receptor (AR) but stained strongly for SV40 T-Ag [23], consistent with primary TRAMP prostate tumors (Fig 2B and 2C). PCR analysis of DNA isolated from liver tumors confirmed that the tumors carried the TRAMP transgene. Since the NSG recipients lacked the transgene, these results confirmed that the tumors originated from TRAMP donor CTCs (Supplementary Fig. S1). In summary, tumor cells were readily identified in blood from all TRAMP animals, but injection of TRAMP blood cells yielded tumors in only 22% of recipients. When tumors did form, they recapitulated the highly invasive and aggressive features of the parental TRAMP tumors. These results indicate that in TRAMP mice, a small minority of CTCs have tumorigenic potential.

### Isolation of viable human prostate cells from human blood

**Figure 3:**
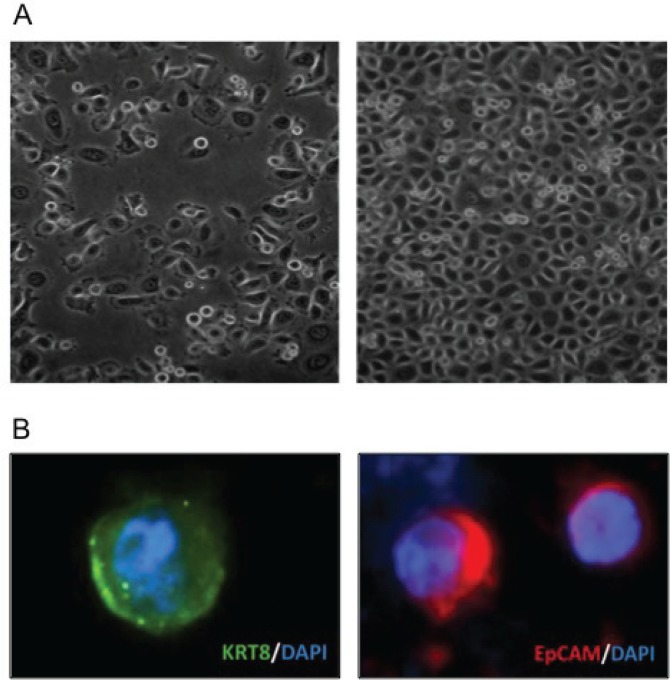
Isolation of tumor cells (A) DU145 spiked in blood recovered using gradient centrifugation were viable 24 hours later (left) and had doubled by 48 hours (right). (B) CTCs isolated from patients' blood express the epithelial markers keratin 8 (left) and EpCAM (right).

As a prerequisite to testing the tumor forming potential of human CTCs, we confirmed that we could isolate viable and growth-competent human prostate cancer cells from human blood. Human DU145 prostate cancer cells were spiked into blood of healthy individuals and recovered using density gradient centrifugation. Upon subsequent culture, these cells expanded at a rate similar to the parental cell line (Fig. 3A). These preliminary findings encouraged us to characterize CTCs present in patients with prostate cancer. Using the same approach, we identified cells expressing the epithelial marker cytokeratin 8 and EpCAM in blood from castration-resistant PCa patients, but not from healthy controls (Fig. 3B and data not shown). These results show that this method recovered viable PCa cells present in human blood.

### Xenografts of human CTCs

**Figure 4:**
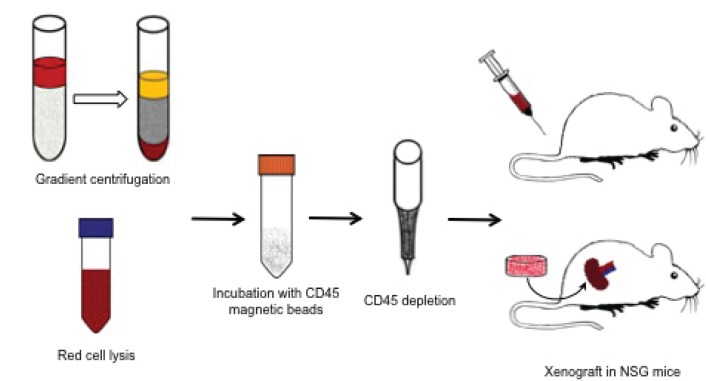
Blood from prostate cancer patients was either placed atop of a Ficoll-Paque PLUS gradient column or subject to red cell lysis; then the nucleated cells were incubated with CD45 magnetic beads and hematopoietic CD45-expressing cells removed from the mixture The remaining cells were either injected into the tail vein of NSG mice or wrapped with mouse neonatal mesenchymal cells and grafted under the kidney capsule.

**Figure 5:**
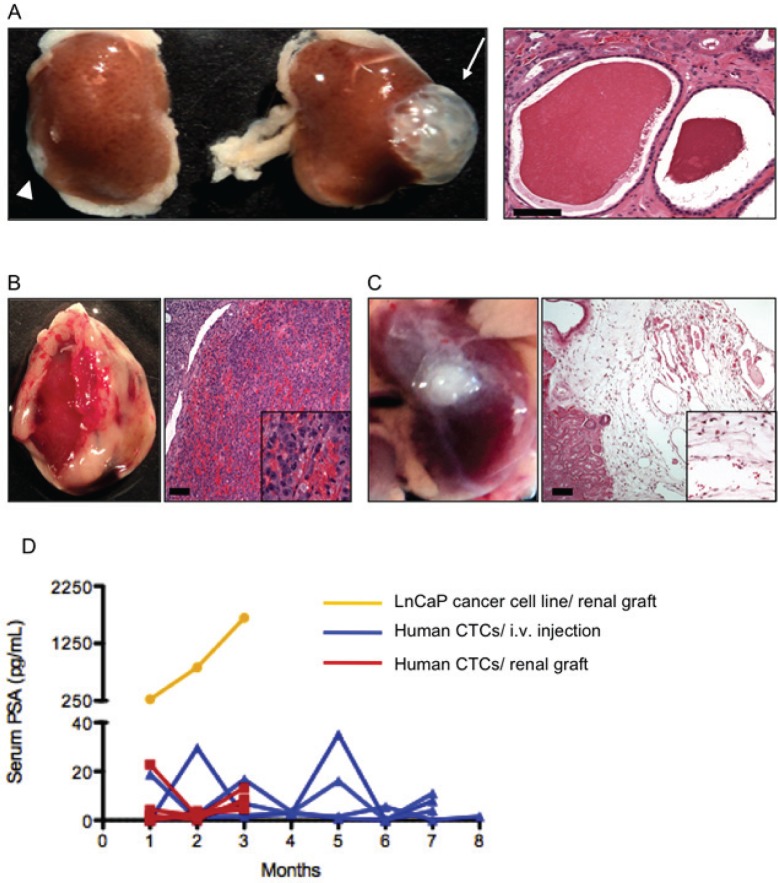
Renal grafts (A) When combined with neonatal murine SVM and grafted in the kidney, benign murine prostate epithelium formed a prostate (arrow and right panel). Scale bar, 100 nm. Neither SVM (not shown) nor prostate epithelial cells alone (arrowhead) did so. (B) Human LnCaP prostate cancer cells engulfed and invaded the kidney. Scale bar, 100 μm. (C) Human CTCs failed to grow and form tumors when grafted under the kidney capsule (left panel). Microscopic analysis of the graft (right panel) revealed many capillary vessels and scattered cells. Scale bar, 100 μm. (D) PSA kinetics in mouse serum reflected these findings: mouse grafted with LnCaP had a rapidly rise in PSA and the hosts of human CTCs showed transient spikes in PSA but not sustained elevation that reflected tumor growth.

After finding metastatic potential of TRAMP CTCs, we decided to inject human CTCs in immunodeficient mice (Fig. 4). Initially, we injected CTCs from PCa patients thought the tail vein of NSG mice (n=4) and measured the PSA in mice serum for 8 months. We observed transient spikes in PSA values, but not sustained elevation that would reflect tumor growth. We euthanized these four mice, performed gross and histologic examination of the viscera, and did not find evidence of engraftment. We then adapted a xenografting method optimized for primary human prostate cancers [17] to combine human CTCs with mouse fetal urogenital mesenchyme and engraft under the kidney capsule. Confirming that the fetal mesenchyme was active and competent to induce prostate growth, murine prostate epithelial cells grew as renal grafts only when recombined with fetal urogenital mesenchyme (Fig. 5A). This grafting method was also highly effective for human prostate cancer cell lines, as shown by grafts combining human LnCaP prostate cancer cells with fetal urogenital mesenchyme. LnCaP grafts engulfed and invaded the kidney (Fig. 5B), resulting in rapidly rising serum PSA in the mouse recipients (Fig. 5D). The seven human CTC grafts introduced under the kidney capsule showed either no rise in PSA or a transient rise followed by return to baseline. Macroscopic and microscopic evaluation of these grafts revealed multiple capillary vessels and scattered cells inside the matrigel matrix without tumor growth (Fig. 5C).

## DISCUSSION

Our results indicate that viable prostate CTCs can be isolated using “negative selection” methods that avoid assumptions regarding size and surface marker expression. When isolated in this way, CTCs can form new tumors. TRAMP CTCs successfully engrafted in 22% of cases and NSG recipients developed in a short period of time small-cell SV40-T Ag positive diffuse liver metastasis.

Using the same methodology on human PCa patients' blood samples, we identified circulating cells expressing EpCAM and cytokeratin 8, the same markers used by Food and Drug Administration (FDA)-cleared automated systems to identify CTCs (CellSearch, Veridex LLC, Raritan, NJ). The identification of these cells confirmed that we were grafting the same cells currently used as biomarkers to predict PCa patients' prognosis [1, 24]. These cells can be introduced into highly immunodeficient mice without adverse events, namely graft-versus-host disease. However, even using highly efficient xenografting techniques [17, 25], human CTCs did not form new tumors. The superior performance of TRAMP CTCs in xenografting assays may have methodological and biological explanations. In the TRAMP experiments, we collected 50% of the blood volume of each mouse, whereas the human sample represented less than 0.5% of blood volume. Therefore, sampling issues should affect human samples more. It is also possible that CTCs have growth requirements that differ from those of primary human prostate cancers. It would thus be possible that the renal capsule/SVM environment is not optimized for CTC growth. For example, the preferential metastatic niche for human prostate cancer is the bone. Bone-based environments might therefore provide better growth support for metastatic PCa, but bone metastasis models have yet to be successfully developed for prostate cancer [26]. Humanized mice represent another option for enhanced xenografting, as they have been shown to increase the take rate of human primary ovarian and lung cancer cells [27-29]. In addition to potential issues with the host environment, we posit that CTCs are not particularly potent at initiating new sites of tumor growth. Previous studies showed that 70% of men with clinically localized PCa undergoing radical prostatectomy have tumor cells in the bone marrow prior to surgery [30]. In the same study, almost 60% of these patients with tumor cells in bone marrow did not have biochemical recurrence after surgery, meaning the majority of the CTCs do not have the capacity to generate metastasis. These results are consistent with our findings that even CTCs isolated from patients with advanced metastatic castration-resistant PCa, did not show the ability to initiate new tumors. If it is true that most prostate CTCs are innocuous, future studies should focus on isolating the few CTCs that can actually promote cancer progression. Isolating CTCs without restrictions regarding size and epithelial differentiation should facilitate such studies.

## MATERIALS AND METHODS

### Patient selection

Patients treated at the Johns Hopkins Sidney Kimmel Comprehensive Cancer Center (Baltimore, MD) with castration-resistant PCa and radiologic evidence of distant osseous or soft tissue metastatic disease were recruited according to an institutional review board (IRB)-approved protocol. All patients signed a written informed consent. A total of 14 patients (Table 1) donated 7 mL of blood on one or more occasions for CTCs immunocytochemistry or xenograft. Blood from healthy individuals without evidence of PCa was spiked with DU145 cells.

**Table 1:** Patients baseline demographics and clinical characteristics

	Overall n=14
**Age, yr, median (IQR)**	69.5 (60 – 81)
	
**Gleason Score, no. (%)**	
Gleason 6	2 (14.3)
Gleason 7	2 (14.3)
Gleason 8	2 (14.3)
Gleason 9	7 (50.0)
Gleason 10	1 (7.1)
	
**Primary Therapy**	
Radical prostatectomy, no. (%)	3 (21.4)
Radiation therapy, no. (%)	3 (21.4)
Combination of prostatectomy and radiation, no. (%)	2 (14.3)
No primary treatment, no. (%)	6 (42.9)
	
**Prior androgen deprivation therapy, no. (%)**	14 (100)
Number of secondary hormonal therapies, median (IQR)	2 (1 − 5)
Three or more hormonal therapies, no. (%)	8 (57.1)
	
**Prior Chemotherapy, no.**	
One prior regimen, no. (%)	9 (64.3)
Two (or more) prior regimens, no. (%)	2 (14.3)
	
**PSA when CTCs when were collected, median (IQR)**	92.9 (3.1 − 4760.2)
	
**Sites of metastases**	
Bone only, no. (%)	5 (35.7%)
Soft tissue only (nodes, liver, lung)	1 (7.1%)
Soft tissue and bone	8 (57.1%)

### Cell Culture

Human DU145 and LnCaP prostate cancer cells were obtained from the American Type Culture Collection, maintained in RPMI 1640 media (Gibco) supplemented with 10% fetal bovine serum (Gibco) and Penicillin-Streptomycin (Invitrogen).

### Cell isolation

Blood drawn into a heparinized tube followed two different approaches (Fig. 1 and 4) to remove red blood cells and collect nucleated cells: red cell lysis or density gradient centrifugation. After red cell removal, nucleated cells present in blood were collected, incubated with anti-leukocyte antibody CD45 magnetic microbeads (Miltenyi Biotec), placed into magnetic depletion columns (Miltenyi Biotec) to deplete white blood cells and all tumor cells that flow through the columns were collected for further studies. As a “proof-of-principle” for isolation of viable circulating cells, human DU145 prostate cancer (1 × 10^6^) cells were spiked in blood from healthy individuals and placed back in culture for 48 hours to monitor if the cells were viable and able to expand in culture. CTCs from PCa patients were either spread across microscopic slides for immunofluorescent analysis or xenografted in mice.

### Generation of CTC xenografts and determination of engraftment with PSA

CTCs from both TRAMP mice and human prostate cancer patients were inserted into anesthetized male highly immunodeficient NOD.Cg-*Prkdc*^*scid*^ *Il2rg*^*tm1Wjl*^/SzJ mice (Figure 1 and 4), also known as NOD SCID gamma (NSG) mice [31]. NSG mice lack functional T cells, B cells, and NK cells and have markedly reduced dendritic cell and macrophage activity. This high degree of immunodeficiency results in superior engraftment of human cells [32].

Approximately 700 mL of blood from TRAMP mice (n=9) went through the red cell lysis step and nucleated cells were afterwards injected into the tail vein of NSG mice. To confirm the existence of CTCs in TRAMPs blood, we spread TRAMP nucleated cells in a microscope slide and performed Wright's stain.

CTCs isolated from PCa patient blood were injected into the tail vein of NSG mice (n=4) or wrapped in mouse newborn seminal vesicle mesenchyme (SVM) and inserted under the renal capsule (n=7) as described previously [17]. Briefly, this technique consists in the isolation of SVM from newborn (day 0) C57BL/6 mice [17], 1 × 10^5^ SVM cells were combined with CTCs isolated from patients in a matrigel (BD Biosciences) disk. The disk, containing CTCs and the SVM were combined and cultured 12 hours in RPMI supplemented with 1 mM of dihydrotestosterone, and then introduced under the renal capsule. To confirm the capacity of SVM to support prostate epithelial growth, benign primary mouse prostate epithelial cells (1 × 10^6^) were combined with SVM and also grafted under the kidney capsule. PSA-producing cancer cells LnCaP (1 × 10^6^) cells were also used as controls, to confirm that the grafting conditions supported human prostate cancer growth and to confirm the performance of ultrasensitive PSA tests (see below) on mouse serum. Since mice do not make PSA, human CTC engraftment was monitored monthly by ultrasensitive nano-PSA assays (Nanosphere Inc, Chicago, IL) performed on serum prepared from 50 mL of mouse blood [33]. NSG mice injected with TRAMP CTCs were euthanized 3 months after grafting, and the NSGs grafted with human circulating cells were euthanized 8 months after grafting. An expert veterinary pathologist performed comprehensive necropsies as well as histologic examination of the liver, lungs, spleen and the grafts from all animals.

### Immunohistochemistry and immunofluorescence

Formalin-fixed, paraffin-embedded tissue samples from the liver, lungs, spleen and CTC xenografts were sectioned (4 mm) and immunohistochemistry was performed as described [34] using anti-SV40 T-Ag (clone Pab101 Santa Cruz, 1:1000) or anti-androgen receptor (clone N-20 Santa Cruz, 1:500) antibodies for immunodetection.

Immunofluorescent staining for cytokeratin 8 (1:500; clone M20 Abcam) and EpCAM (1:500; clone VU-1D9 Abcam) was performed to confirm the collection of CTCs from castration-resistant PCa patients. Cells obtained after CD45 depletion were spread onto a microscope slide, stained as previously described [35] and analyzed under a 100x oil immersion objective using a Nikon E400 fluorescence microscope (Nikon).

## CONCLUSIONS

These results demonstrate that viable CTCs can be isolated without *a priori* assumptions about surface markers and, using transgenic mouse models of PCa cancer, CTCs were able to form metastasis in new hosts. Nevertheless, CTCs were surprising inefficient at initiating new sites of tumor growth. Our work highlights the need to better define subpopulations of CTCs that have the ability to produce metastasis. Future studies should identify and target the highly tumorigenic cells.

## Supplementary Figures



## References

[1] Scher HI, Jia X, de Bono JS, Fleisher M, Pienta KJ, Raghavan D, Heller G (2009). Circulating tumour cells as prognostic markers in progressive, castration-resistant prostate cancer: a reanalysis of IMMC38 trial data. Lancet Oncol.

[2] Strijbos MH, Gratama JW, Schmitz PI, Rao C, Onstenk W, Doyle GV, Miller MC, de Wit R, Terstappen LW, Sleijfer S Circulating endothelial cells, circulating tumour cells, tissue factor, endothelin-1 and overall survival in prostate cancer patients treated with docetaxel. Eur J Cancer.

[3] Patel AS, Allen JE, Dicker DT, Peters KL, Sheehan JM, Glantz MJ, El-Deiry WS (2011). Identification and enumeration of circulating tumor cells in the cerebrospinal fluid of breast cancer patients with central nervous system metastases. Oncotarget.

[4] Faltas B (2011). Circulating tumor cells in the cerebrospinal fluid: “tapping” into diagnostic and predictive potential. Oncotarget.

[5] Danila DC, Fleisher M, Scher HI Circulating tumor cells as biomarkers in prostate cancer. Clin Cancer Res.

[6] Danila DC, Anand A, Sung CC, Heller G, Leversha MA, Cao L, Lilja H, Molina A, Sawyers CL, Fleisher M, Scher HI TMPRSS2-ERG status in circulating tumor cells as a predictive biomarker of sensitivity in castration-resistant prostate cancer patients treated with abiraterone acetate. Eur Urol.

[7] Wang ZP, Eisenberger MA, Carducci MA, Partin AW, Scher HI, Ts'o PO (2000). Identification and characterization of circulating prostate carcinoma cells. Cancer.

[8] Al-Hajj M, Wicha MS, Benito-Hernandez A, Morrison SJ, Clarke MF (2003). Prospective identification of tumorigenic breast cancer cells. Proc Natl Acad Sci U S A.

[9] He X, Marchionni L, Hansel DE, Yu W, Sood A, Yang J, Parmigiani G, Matsui W, Berman DM (2009). Differentiation of a highly tumorigenic basal cell compartment in urothelial carcinoma. Stem Cells.

[10] Kirby BJ, Jodari M, Loftus MS, Gakhar G, Pratt ED, Chanel-Vos C, Gleghorn JP, Santana SM, Liu H, Smith JP, Navarro VN, Tagawa ST, Bander NH, Nanus DM, Giannakakou P Functional characterization of circulating tumor cells with a prostate-cancer-specific microfluidic device. PLoS One.

[11] Huang Z, Hurley PJ, Simons BW, Marchionni L, Berman DM, Ross AE, Schaeffer EM (2012). Sox9 is required for prostate development and prostate cancer initiation. Oncotarget.

[12] Vainio P, Lehtinen L, Mirtti T, Hilvo M, Seppanen-Laakso T, Virtanen J, Sankila A, Nordling S, Lundin J, Rannikko A, Oresic M, Kallioniemi O, Iljin K (2011). Phospholipase PLA2G7, associated with aggressive prostate cancer, promotes prostate cancer cell migration and invasion and is inhibited by statins. Oncotarget.

[13] Poczatek RB, Myers RB, Manne U, Oelschlager DK, Weiss HL, Bostwick DG, Grizzle WE (1999). Ep-Cam levels in prostatic adenocarcinoma and prostatic intraepithelial neoplasia. J Urol.

[14] Mani SA, Guo W, Liao MJ, Eaton EN, Ayyanan A, Zhou AY, Brooks M, Reinhard F, Zhang CC, Shipitsin M, Campbell LL, Polyak K, Brisken C, Yang J, Weinberg RA (2008). The epithelial-mesenchymal transition generates cells with properties of stem cells. Cell.

[15] Brennen WN, Chen S, Denmeade SR, Isaacs JT (2013). Quantification of Mesenchymal Stem Cells (MSCs) at Sites of Human Prostate Cancer. Oncotarget.

[16] Jimeno A, Feldmann G, Suarez-Gauthier A, Rasheed Z, Solomon A, Zou GM, Rubio-Viqueira B, Garcia-Garcia E, Lopez-Rios F, Matsui W, Maitra A, Hidalgo M (2009). A direct pancreatic cancer xenograft model as a platform for cancer stem cell therapeutic development. Mol Cancer Ther.

[17] Toivanen R, Berman DM, Wang H, Pedersen J, Frydenberg M, Meeker AK, Ellem SJ, Risbridger GP, Taylor RA Brief report: a bioassay to identify primary human prostate cancer repopulating cells. Stem Cells.

[18] Zhao H, Nolley R, Chen Z, Peehl DM Tissue slice grafts: an in vivo model of human prostate androgen signaling. Am J Pathol.

[19] Priolo C, Agostini M, Vena N, Ligon AH, Fiorentino M, Shin E, Farsetti A, Pontecorvi A, Sicinska E, Loda M Establishment and genomic characterization of mouse xenografts of human primary prostate tumors. Am J Pathol.

[20] Greenberg NM, DeMayo F, Finegold MJ, Medina D, Tilley WD, Aspinall JO, Cunha GR, Donjacour AA, Matusik RJ, Rosen JM (1995). Prostate cancer in a transgenic mouse. Proc Natl Acad Sci U S A.

[21] Gingrich JR, Barrios RJ, Morton RA, Boyce BF, DeMayo FJ, Finegold MJ, Angelopoulou R, Rosen JM, Greenberg NM (1996). Metastatic prostate cancer in a transgenic mouse. Cancer Res.

[22] Gingrich JR, Barrios RJ, Kattan MW, Nahm HS, Finegold MJ, Greenberg NM (1997). Androgen-independent prostate cancer progression in the TRAMP model. Cancer Res.

[23] Chiaverotti T, Couto SS, Donjacour A, Mao JH, Nagase H, Cardiff RD, Cunha GR, Balmain A (2008). Dissociation of epithelial and neuroendocrine carcinoma lineages in the transgenic adenocarcinoma of mouse prostate model of prostate cancer. Am J Pathol.

[24] Danila DC, Heller G, Gignac GA, Gonzalez-Espinoza R, Anand A, Tanaka E, Lilja H, Schwartz L, Larson S, Fleisher M, Scher HI (2007). Circulating tumor cell number and prognosis in progressive castration-resistant prostate cancer. Clin Cancer Res.

[25] Taylor RA, Toivanen R, Frydenberg M, Pedersen J, Harewood L, Collins AT, Maitland NJ, Risbridger GP, Australian Prostate Cancer B Human epithelial basal cells are cells of origin of prostate cancer, independent of CD133 status. Stem Cells.

[26] Singh AS, Macpherson GR, Price DK, Schimel D, Figg WD (2006). Evaluation of human fetal bone implants in SCID mice as a model of prostate cancer bone metastasis. Oncol Rep.

[27] Bankert RB, Balu-Iyer SV, Odunsi K, Shultz LD, Kelleher RJ, Barnas JL, Simpson-Abelson M, Parsons R, Yokota SJ Humanized mouse model of ovarian cancer recapitulates patient solid tumor progression, ascites formation, and metastasis. PLoS One.

[28] Simpson-Abelson MR, Sonnenberg GF, Takita H, Yokota SJ, Conway TF, Kelleher RJ, Shultz LD, Barcos M, Bankert RB (2008). Long-term engraftment and expansion of tumor-derived memory T cells following the implantation of non-disrupted pieces of human lung tumor into NOD-scid IL2Rgamma(null) mice. J Immunol.

[29] Richmond A, Su Y (2008). Mouse xenograft models vs GEM models for human cancer therapeutics. Dis Model Mech.

[30] Ellis WJ, Pfitzenmaier J, Colli J, Arfman E, Lange PH, Vessella RL (2003). Detection and isolation of prostate cancer cells from peripheral blood and bone marrow. Urology.

[31] Shultz LD, Lyons BL, Burzenski LM, Gott B, Chen X, Chaleff S, Kotb M, Gillies SD, King M, Mangada J, Greiner DL, Handgretinger R (2005). Human lymphoid and myeloid cell development in NOD/LtSz-scid IL2R gamma null mice engrafted with mobilized human hemopoietic stem cells. J Immunol.

[32] Carreno BM, Garbow JR, Kolar GR, Jackson EN, Engelbach JA, Becker-Hapak M, Carayannopoulos LN, Piwnica-Worms D, Linette GP (2009). Immunodeficient mouse strains display marked variability in growth of human melanoma lung metastases. Clin Cancer Res.

[33] Thaxton CS, Elghanian R, Thomas AD, Stoeva SI, Lee JS, Smith ND, Schaeffer AJ, Klocker H, Horninger W, Bartsch G, Mirkin CA (2009). Nanoparticle-based bio-barcode assay redefines “undetectable” PSA and biochemical recurrence after radical prostatectomy. Proc Natl Acad Sci U S A.

[34] Kleeberger W, Bova GS, Nielsen ME, Herawi M, Chuang AY, Epstein JI, Berman DM (2007). Roles for the stem cell associated intermediate filament Nestin in prostate cancer migration and metastasis. Cancer Res.

[35] Outeiro TF, Putcha P, Tetzlaff JE, Spoelgen R, Koker M, Carvalho F, Hyman BT, McLean PJ (2008). Formation of toxic oligomeric alpha-synuclein species in living cells. PLoS One.

