# Supplementary material for: Tumorigenic potential of circulating prostate tumor cells
Source: Oncotarget. 2013 Mar 5;4(3):413–21. doi: 10.18632/oncotarget.895 (PMC3717304; doi:10.18632/oncotarget.895)
Supplement: Supplementary file 1 [file oncotarget-04-413-s001.pdf]

Tumorigenic potential of circulating prostate tumor cells - Carvalho et al

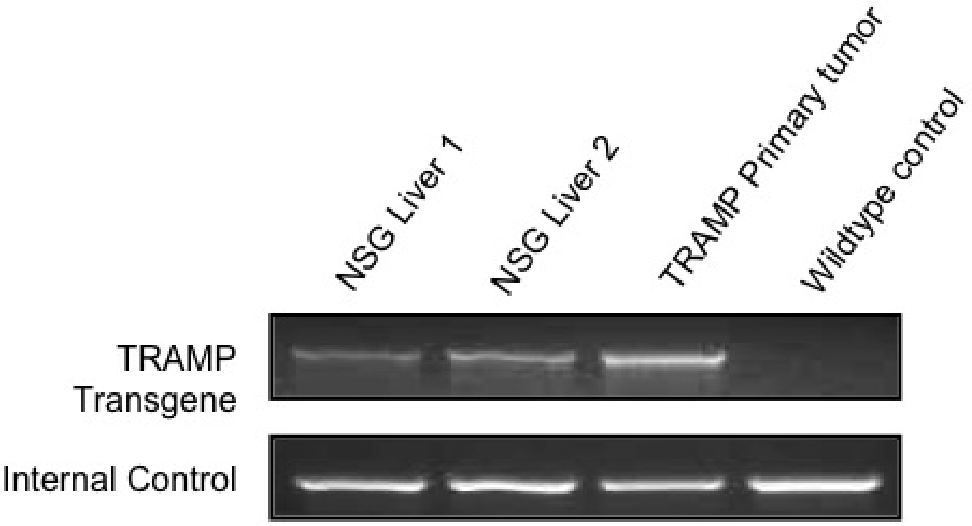

Supplementary figure S1: PCR analysis of genomic DNA isolated from liver tumors. PCR product of TRAMP transgene was detected in the liver of NSG mice with metastasis, confirming the tumors originated from TRAMP tumor.
